# P-64. Assessing Outcomes and Methods of Early Switch to Oral Therapy for Low-Risk Staphylococcus aureus Bacteremia: A Retrospective Chart Review Study

**DOI:** 10.1093/ofid/ofaf695.293

**Published:** 2026-01-11

**Authors:** Nikita Shah, Maximillian S Wu, Vagdevi Seetamraju, Nabil Zeineddine

**Affiliations:** SUNY Upstate Medical University, Syracuse, NY; SUNY Upstate Medical University, Syracuse, NY; SUNY Upstate Medical University, Syracuse, NY; SUNY Upstate Medical University, Syracuse, NY

## Abstract

**Background:**

The current standard of care for low-risk *Staphylococcus aureus* bacteremia (SAB) involves two weeks of intravenous (IV) antibiotic therapy. However, emerging evidence suggests that transitioning to oral antibiotics during the treatment course may be safe and effective. The SABATO randomized controlled trial notably demonstrated that early oral step-down therapy was non-inferior to continued IV therapy in low-risk SAB cases.

**Methods:**

We retrospectively reviewed adult patients diagnosed with SAB at our institution in 2023. Patients were included if they met criteria for low-risk SAB and completed a two-week course of antibiotic therapy. Patients were categorized into two groups: those who received IV therapy exclusively and those who transitioned to oral antibiotics. Clinical outcomes and adverse events were assessed within 90 days from the initiation of treatment.

**Results:**

Out of 173 patients evaluated, 45 met criteria for low-risk SAB. 12 patients received IV antibiotics exclusively, while 33 patients transitioned to and completed treatment with oral agents. The most commonly prescribed oral antibiotics were linezolid 600 mg BID (n=24), cephalexin 1g TID (n=4), and trimethoprim-sulfamethoxazole DS BID (n=2). Adverse events occurred in 50% (n=6) of the IV-only group and 48.5% (n=16) in the oral group (p=0.928). All-cause mortality was 0% in the IV group and 9% (n=3) in the oral group. *Clostridioides difficile* colitis occurred in 8% (n=1) of the IV group and 6% (n=2) of the oral group. Recurrent SAB was observed in 3% (n=1) of the oral group and 0% of the IV group.

**Conclusion:**

In this single center cohort of patients with low-risk SAB, partial oral antibiotic therapy was not associated with a statistically significant increase in adverse events compared to exclusive IV therapy. While all-cause mortality was higher in the oral therapy group, the small sample size limits definitive conclusions. These findings support the growing body of evidence that oral step-down therapy may be a viable option in selected cases of low-risk SAB.

**Disclosures:**

All Authors: No reported disclosures

